# Avaliação Pré-Participação no Futebol: Um Pilar Estratégico para as Copas de 2026 e 2027

**DOI:** 10.36660/abc.20260084

**Published:** 2026-05-29

**Authors:** Ricardo Stein

**Affiliations:** 1 Universidade Federal do Rio Grande do Sul Porto Alegre RS Brasil Universidade Federal do Rio Grande do Sul, Porto Alegre, RS – Brasil

**Keywords:** Futebol, Triagem, Atletas

No horizonte desportivo imediato, dois eventos de grande magnitude irão redefinir as exigências do futebol de alto rendimento: a Copa do Mundo da FIFA 2026™, com seu formato expandido e uma maratona física sem precedentes, e a Copa do Mundo Feminina da FIFA 2027™, a ser realizada no Brasil, símbolo da consolidação da modalidade. Nesse contexto, a avaliação pré-participação (APP) do futebolista deixa de ser apenas um protocolo clínico obrigatório e passa a assumir papel estratégico central para o desempenho desportivo e a sustentabilidade das carreiras atléticas.

A Copa de 2026, em particular, impõe um desafio extremo: 48 seleções, 104 jogos, deslocamentos continentais entre Estados Unidos, Canadá e México, além de um calendário condensado. Trata-se, sobretudo, de uma prova de resistência sem precedentes na história do torneio. Diante desse cenário, a APP não pode se restringir ao tradicional *check-up* cardiológico e ortopédico. É necessário avançar para um modelo contínuo e integrado de monitorização, que inclua análise detalhada da carga de trabalho nos últimos 12 meses, com o objetivo de identificar atletas em limiar de sobrecarga; avaliação da resiliência mental e da tolerância ao estresse, fatores decisivos em competições prolongadas e de alta pressão; e utilização de monitorização biomolecular para detecção precoce de fadiga acumulada e risco de lesão. Essa abordagem proativa configura a primeira linha de defesa na preparação do atleta para uma "maratona" em que a integridade física será tão determinante quanto a técnica.

Essa perspectiva também se relaciona à proteção do patrimônio esportivo. Federações investem recursos expressivos em suas seleções, e a perda inesperada de um jogador-chave, seja por condição cardíaca subjacente não diagnosticada ou por lesão muscular recorrente mal manejada, representa não apenas uma tragédia individual, mas também um impacto esportivo e financeiro significativo. Sob essa ótica, a APP, no contexto da Copa do Mundo da FIFA, seja masculina ou feminina, funciona como um mecanismo essencial de proteção do investimento, resguardando tanto o atleta, patrimônio humano de difícil substituição, quanto a equipe, enquanto patrimônio esportivo coletivo.

No caso da Copa do Mundo Feminina da FIFA 2027™, a APP assume ainda maior relevância simbólica e prática. O evento representa uma oportunidade histórica para consolidar uma cultura de cuidado específico no futebol feminino, com a implementação de protocolos que considerem as particularidades das atletas. Isso inclui atenção ao risco aumentado de determinadas lesões, como as rupturas do ligamento cruzado anterior; incorporação da fisiologia do ciclo menstrual na gestão da performance e do risco lesivo;^1^ e garantia de que a triagem cardiovascular seja conduzida com o mesmo rigor aplicado aos atletas masculinos, corrigindo uma lacuna histórica de subinvestigação. Um programa de APP robusto em 2027 tem potencial não apenas para aumentar as chances de desempenho competitivo em nível máximo, mas também para estabelecer um legado duradouro de equidade na medicina do esporte.

Naturalmente, esse processo não está isento de dilemas éticos.^2^ Surge, então, a questão de como proceder quando a APP identifica um risco moderado em um atleta considerado indispensável. O conflito entre o dever de cuidado e a pressão competitiva atinge seu ponto máximo em ano de Copa. Nesses casos, a solução deve basear-se em tomada de decisão multidisciplinar e transparente, envolvendo médicos, fisioterapeutas, treinadores e o próprio atleta, sempre fundamentada em evidências científicas e orientada pelo princípio inegociável do bem-estar a longo prazo.

O estabelecimento de um padrão mínimo global pela FIFA constitui uma responsabilidade institucional essencial para garantir o rigor da APP. Esse princípio está em processo de consolidação por meio da elaboração de documentos como o *FIFA Consensus on Recommendations for Cardiac Screening of Adult Football Players* (no prelo), do qual sou um dos autores. Participei do grupo de especialistas reunido em Zurique, Suíça, nos dias 5 e 6 de fevereiro de 2026, com o objetivo de refinar e consolidar os conhecimentos previamente publicados sobre jogadores com < 18 anos,^3^ desta vez com foco em futebolistas com ≥ 18 anos.

Entretanto, a efetividade dessa padronização depende da sua capacidade de acomodar diferentes realidades. Atletas de distintas origens, como noruegueses, argentinos e japoneses, apresentam históricos de carga, perfis nutricionais e exposições ambientais significativamente distintos. Por esse motivo, a aplicação do padrão global não deve ser rígida. A APP ideal deve combinar rigor científico universal com adaptação prática local, ajustando-se às características específicas de cada atleta e ao contexto desportivo nacional.

Considera-se que uma anamnese detalhada, um exame físico minucioso e o eletrocardiograma (ECG) de repouso de 12 derivações constituem o conjunto fundamental para o início de qualquer processo de APP. Esse núcleo básico representa a base para a detecção de condições silenciosas, especialmente cardiovasculares. Reconhecendo a importância do ECG e a necessidade de dados específicos da população brasileira, nosso grupo de pesquisa, em colaboração com especialistas em Medicina do Esporte e Cardiologia de diferentes regiões do país, tem conduzido investigações sistemáticas sobre a interpretação do ECG em atletas de futebol no Brasil.

Como resultado desse esforço colaborativo, foram publicados quatro artigos científicos em periódicos internacionais indexados, que forneceram, pela primeira vez em escala nacional, dados de referência de ECG e a prevalência de achados fora do padrão habitual em uma ampla coorte de jogadores profissionais masculinos das séries A-D filiados à Confederação Brasileira de Futebol.^4–7^ Essa produção científica nacional é fundamental para calibrar os critérios internacionais de interpretação,^8^ permitindo que a triagem cardiovascular no Brasil seja realizada com base em evidências locais. Dessa forma, reduz-se o risco de diagnósticos falso-positivos, que podem afastar injustamente atletas saudáveis, e, ao mesmo tempo, evita-se a subestimação de achados clinicamente relevantes para a realidade nacional.

Em conclusão, a APP para as Copas de 2026 e 2027 não deve ser tratada como um simples procedimento burocrático. Trata-se de um componente estratégico, que deve ser compreendido como o primeiro movimento tático de qualquer seleção que almeja o título. Quando aplicada com rigor científico e uma abordagem holística, a APP permite alcançar três objetivos centrais: i) maximizar o desempenho, ii) reduzir riscos e iii) promover uma abordagem mais humanizada do alto rendimento.

O legado dessas Copas deve ser avaliado não apenas pelos momentos de excelência dentro de campo, mas também pela consolidação de um novo paradigma de cuidado integral com a saúde do atleta. Em um cenário no qual a diferença entre vitória e frustração pode residir em detalhes mínimos, a equipe mais bem-sucedida poderá ser aquela que chegar às fases finais com seu elenco mais saudável, preparado e protegido. Essa trajetória começa, inevitavelmente, com uma APP bem conduzida.

## Data Availability

Os conteúdos subjacentes ao texto da pesquisa estão contidos no manuscrito.
