# Supplementary material for: Avaliação Pré-Participação no Futebol: Um Pilar Estratégico para as Copas de 2026 e 2027
Source: Arq Bras Cardiol. 2026 May 29;123(4):e20260084. [Article in Portuguese] doi: 10.36660/abc.20260084 (PMC13398886; doi:10.36660/abc.20260084)
Supplement: Translation

# Translation: Pre-Participation Evaluation in Football: A Strategic Pillar for the 2026 and 2027 World Cups

**DOI:** 10.36660/abc.20260084i

**Authors:** Ricardo Stein

**Affiliations:** 1 Porto Alegre RS Brazil Universidade Federal do Rio Grande do Sul, Porto Alegre, RS – Brazil

**Keywords:** Soccer, Triage, Athletes

Two major events on the immediate sporting horizon will redefine the demands of high-performance football: the expanded format of the FIFA World Cup 2026™ and the FIFA Women's World Cup Brazil 2027™, which symbolizes the consolidation of the sport. In this context, the pre-participation evaluation (PPE) of footballers is no longer just a mandatory clinical protocol, but rather, it plays a central strategic role in athletic performance and career sustainability.

In particular, the 2026 World Cup presents an extreme challenge: 48 teams, 104 matches, and continental travel across the United States, Canada, and Mexico, all within a condensed schedule. Above all, the tournament will be a test of endurance unlike anything in its history. Thus, PPE cannot be limited to traditional cardiological and orthopedic checkups. A continuous, integrated monitoring model is necessary and should include a detailed analysis of the athlete's workload over the previous 12 months. This analysis aims to identify athletes at the threshold of overload. It also includes an assessment of mental resilience and stress tolerance, which are decisive factors in prolonged, high-pressure competitions. Additionally, biomolecular monitoring is necessary for the early detection of accumulated fatigue and injury risk. This proactive approach is the first line of defense in preparing athletes for a "marathon" where physical integrity is as important as technical ability.

This perspective is also related to protecting sporting assets. Federations invest significant resources in their national teams. The unexpected loss of a key player due to an undiagnosed cardiac condition or a poorly managed muscle injury represents an individual tragedy and has significant sporting and financial implications. PPE thereby functions as an essential mechanism for protecting investment and safeguarding both the athlete, who is a difficult-to-replace human asset, and the team, which is a collective sporting asset, in the context of the FIFA World Cup, whether men's or women's.

For the FIFA Women's World Cup Brazil 2027™, PPE takes on an even greater symbolic and practical importance. It provides a historic opportunity to establish a culture of personalized care in women's football by implementing protocols that address the unique needs of female athletes, which includes addressing the increased risk of certain injuries, such as anterior cruciate ligament ruptures, and incorporating menstrual cycle physiology into performance management and injury risk.^1^ It also includes ensuring that cardiovascular screening is conducted with the same rigor applied to male athletes, thus correcting a historical underassessment gap. A robust PPE program in 2027 could increase the likelihood of peak performance and establish a lasting legacy of equity in sports medicine.

Naturally, this process is not without ethical dilemmas.^2^ The question then arises of how to proceed when PPE identifies a moderate risk in an indispensable athlete. The conflict between the duty of care and competitive pressure is at its height in a World Cup year. In such cases, the solution must be based on transparent, multidisciplinary decision-making involving physicians, physical therapists, coaches, and the athlete. This process must always be grounded in scientific evidence and guided by the non-negotiable principle of long-term well-being.

FIFA's establishment of a global minimum standard is an essential institutional responsibility that ensures the rigor of PPE. This principle is currently being solidified through the development of documents such as the forthcoming FIFA Consensus on Recommendations for Cardiac Screening of Adult Football Players (in press), of which I am one of the authors. I participated in the expert group convened in Zurich, Switzerland, on February 5 and 6, 2026, which aimed to refine and consolidate previously published knowledge on players < 18 years of age,^3^ this time focusing on footballers aged ≥ 18 years.

However, the effectiveness of this standardization depends on its ability to accommodate different realities. For example, athletes from different backgrounds, such as Norwegians, Argentinians, and Japanese players, have significantly different histories of workloads, nutritional profiles, and environmental exposures. Therefore, the application of a global standard should be flexible. Optimal PPE combines universal scientific rigor with local practical adaptation, adjusting to each athlete's specific characteristics and the national sporting context.

A detailed medical history, thorough physical examination, and resting 12-lead electrocardiogram (ECG) are considered the fundamental components of initiating any PPE process. This foundation is essential for identifying silent conditions, particularly cardiovascular ones. Recognizing the importance of the ECG and the need for population-specific data in Brazil, our research group has been conducting systematic investigations on ECG interpretation in Brazilian football players in collaboration with Sports Medicine and Cardiology experts from different regions of the country.

As a result of this collaborative effort, four scientific articles have been published in indexed international journals. For the first time on a national scale, these articles provide reference ECG data and the prevalence of out-of-the-ordinary findings in a large cohort of male professional players from divisions A-D affiliated with the Brazilian Football Association.^4–7^ This national scientific output is essential for calibrating international interpretation criteria,^8^ which allows cardiovascular screening in Brazil to be conducted based on local evidence. This reduces the risk of false-positive diagnoses that may unjustly exclude healthy athletes while avoiding the underestimation of clinically relevant findings specific to the national context.

In conclusion, PPE for the 2026 and 2027 World Cups should not be treated as a mere bureaucratic procedure. Rather, it is a strategic component that must be understood as the first tactical move of any national team aiming for the title. When applied with scientific rigor and a holistic approach, PPE enables the achievement of three key objectives: i) maximizing performance, ii) reducing risks, and iii) promoting a more humanized approach to high-performance sports.

The legacy of these World Cups should be evaluated based not only on moments of excellence on the field, but also on the establishment of a new model of comprehensive athletic health care. In a scenario where victory or frustration may hinge on minimal details, the most successful team may be the one that reaches the final stages with a healthier, better-prepared, better-protected roster. This journey inevitably begins with a thorough PPE.

## Data Availability

The underlying content of the research text is contained within the manuscript.
